# Peritoneal metastases from colorectal cancer managed by stereotactic body radiation therapy: presentation of a clinical case and review of literature

**DOI:** 10.1093/bjrcr/uaaf060

**Published:** 2025-12-08

**Authors:** Alexander Bennassi, Stanislas Ropert, Gokoulakrichenane Loganadane, Clarisse Dromain, Toufik Bennassi

**Affiliations:** Department of Radio-Oncology, Centre de Cancérologie de Thiais, Thiais 94320, France; Department of Oncology, Hôpital Privé d’Antony, Antony 92160, France; Department of Radiation Oncology, Institut Curie, Paris 75005, France; Department of Radiology, Centre Hospitalier Universitaire Vaudois, Lausanne 1011, Switzerland; Department of Radio-Oncology, Centre de Cancérologie de Thiais, Thiais 94320, France

**Keywords:** peritoneal metastases, oligorecurrent, stereotactic body radiation therapy

## Abstract

Peritoneal metastasis (PM, or peritoneal carcinomatosis) is often associated with dismal prognosis. The treatment of peritoneal metastasis depends on several factors, including the type of primary cancer, the extent of metastasis and the patient’s overall health condition. Apart from systemic chemotherapy and cytoreductive surgery (CRS), locoregional therapies such as hyperthermic intraperitoneal chemotherapy (HIPEC) or pressurized intraperitoneal aerosol chemotherapy (PIPAC) may improve tumour control. CRS is a complex procedure with high morbidity, performed only in approximately 25% of all eligible patients and the efficiency of other non-surgical therapies is not well known. The place of radiation therapy needs to be defined. A 62-year-old woman was referred for surgery for a few days’ history of large bowel obstruction. A previous abdominopelvic CT showed an obstructive 3-cm mass in the proximal ascending colon, associated with small bowel dilatation. Right hemi-colectomy was performed. Work-up demonstrated on MRI (magnetic resonance imaging) sub-centimetric secondary liver lesions. ^18^F-fluorodeoxyglucose positron emission tomography/computed tomography (PET/CT) ruled out other metastatic lesions. After multidisciplinary discussion, peri-operative systemic therapy based on FOLFOX-Bevacizumab was offered and was associated with stable disease for 7 months. Unfortunately, peritoneal progression was detected on PET/CT imaging. FOLFIRI-Bevacizumab was offered as second line systemic therapy followed by resection of residual masses. The patient was considered in remission for 5 months thereafter. A second isolated peritoneal relapse occurred involving the same 2 distinct sites. Second line chemotherapy was re-challenged. The oligorecurrent PM lesions were considered as non-operable. After multidisciplinary team discussion, stereotactic body radiation therapy (SBRT) was decided. The relapse sites were treated with 48 Gy, delivered in 6 fractions of 8 Gy given every other day. At the 22-month follow-up, the patient showed no signs of relapse on CT imaging and no treatment-related toxicities. Isolated peritoneal oligometastatic metastases are associated with poor prognosis. Cytoreductive surgery remains the historical standard of care treatment but represents a minority of all eligible patients. Our case and few other reports suggest that SBRT is a safe, reasonable, and non-invasive treatment option.

## Introduction

Peritoneal metastasis (PM) are, after the liver, the second most common site of recurrence of colon cancer and the second cause of death in patients with colorectal cancer.^1^^,^^2^ Prognosis is commonly very poor, with median overall survival (OS) of 15 to 24 months despite systemic chemotherapy.^3^

Cytoreductive surgery (CRS) combined with or without hyperthermic intraperitoneal chemotherapy (HIPEC) has yielded a significant improvement in patients with colorectal PM with a median OS of 41.45 months (range, 41.2-41.7 months).^4^^,^^5^ In stage III epithelial ovarian cancer, the addition of HIPEC has demonstrated to prolong both recurrence-free survival and overall survival.^6^ However, this is a complex procedure with high morbidity, performed only in approximately 25% of all eligible patients.^1^ Effective treatment of non-operable patient is lacking.^1^

The consideration of PM as a locoregional rather than a metastatic disease combined to the emerging state of oligometastatic disease (OMD) have changed the management paradigm of patients with PM from a palliative perspective to a more tumour-based approach (with targeted and local therapies) in a curative intent.^2^^,^^7^ Although surgery is the preferred treatment of oligometastatic peritoneal disease, an increasing number of minimal invasive local therapies have emerged. Stereotactic body radiation therapy (SBRT) has gained popularity in recent years, due to its advantages such as non-invasive ablative treatment, shorter recovery time, and low complications rates.^2^

We hereby report a case of an oligorecurrent PM from colorectal cancer, managed by SBRT. To the best of our knowledge, it is the first reported case with oligorecurrent PM from colorectal cancer managed by SBRT using volumetric-modulated arc therapy (VMAT) technique.

## Case report

A 62-year-old woman was referred for surgery after persistent symptomatic obstructive syndrome. She had no prior history of abdominal surgery nor family history of cancer. Contrast-enhanced abdominopelvic CT images showed an obstructive 3-cm mass in the proximal ascending colon, associated with small bowel dilatation.

Right hemi-colectomy was performed in July 2018, and pathology revealed a pT3 pN0 (0/23) G1 R0, Pn1, L0, V0, microsatellite stable (MSS) adenocarcinoma of the right colon. The work-up demonstrated on MRI 3 sub-centimetric secondary liver lesions (segment VI, VII and segment VII/VIII) on MRI (magnetic resonance imaging). ^18^F-fluorodeoxyglucose positron emission tomography/computed tomography (PET/CT) found no additional lesion. Molecular findings from the surgical specimen found a KRAS mutation involving the exon 2 c.35 G > A and no NRAS and BRAF mutations. The multidisciplinary tumour board suggested a peri-operative systemic approach with Folfox-Bevacizumab.

The patient had a partial response and underwent right hepatectomy. Histology revealed a single residual 5-mm metastasis of the right liver, with subcapsular infiltration. Systemic treatment was resumed, and the patient had a stable disease for 7 months.

**Figure 1.**
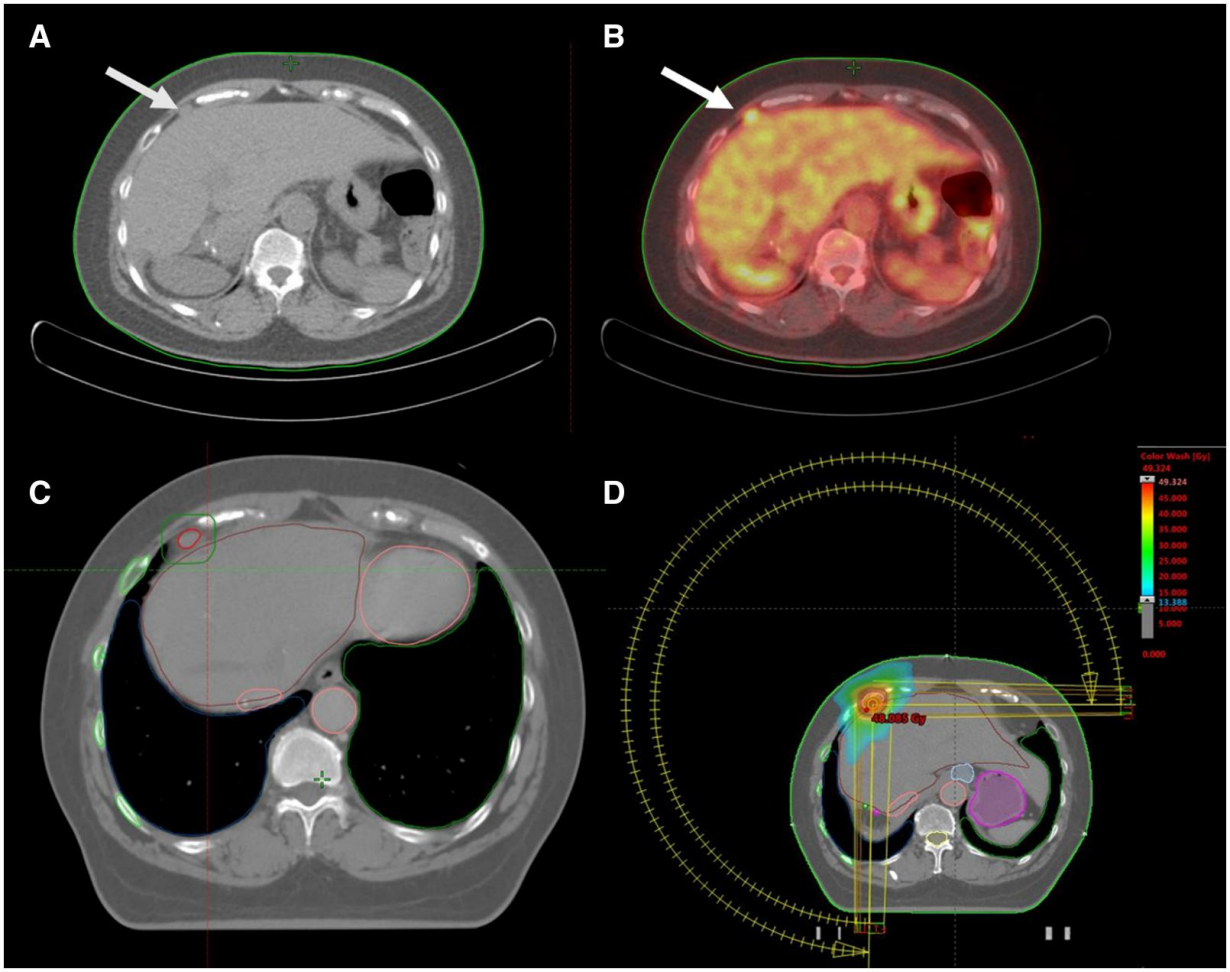
Peritoneal metastasis (hepatic dome) managed by SBRT: radiologic and dosimetric features. (A) Unenhanced CT image demonstrating peritoneal metastasis (arrow) regarding the hepatic dome. (B) ^18^F-fluorodeoxyglucose positron emission tomography/computed tomography (PET/CT) image showing tracer uptake of the peritoneal metastasis. (C) Unenhanced CT-scan with the gross tumour volume (GTV) encompassing the lesion. The planning target volume (PTV) covered the GTV with 5 mm margin. The treatment was performed with 6 megavoltage (MV) flattening filter free (FFF) photons on a linear accelerator using VMAT technique. (D) VMAT plan showing axial images of radiotherapy dose colour wash, with a hot spot (48.085 Gy) within the PTV.

**Figure 2.**
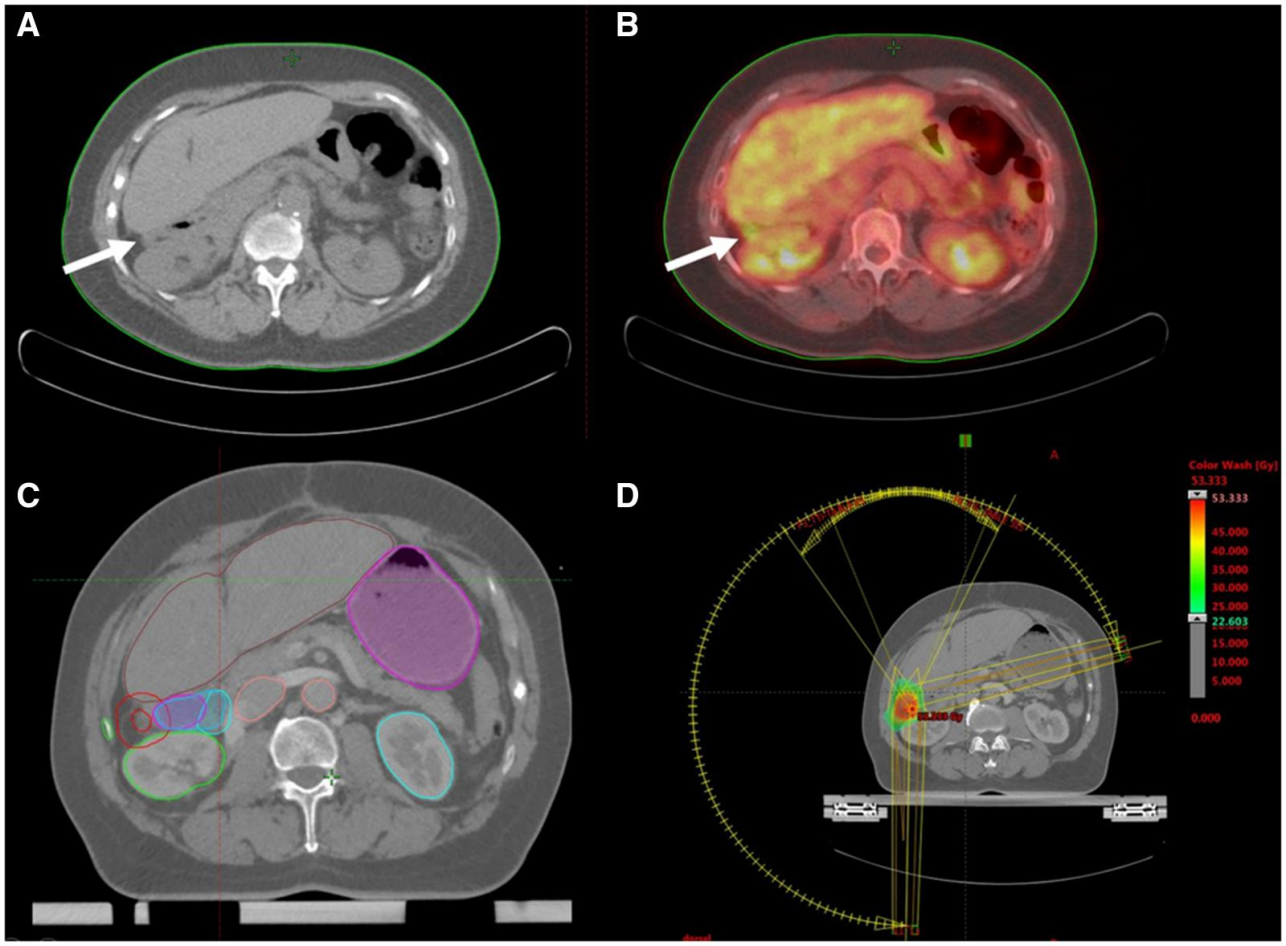
Peritoneal metastasis (right Morrison pouch) managed by SBRT: radiologic and dosimetric features. (A) Unenhanced CT image demonstrating peritoneal metastasis in the right Morrison pouch. (B) ^18^F-fluorodeoxyglucose positron emission tomography/computed tomography (PET/CT) showing uptake of the peritoneal metastasis. (C) Unenhanced CT-scan with the gross tumour volume (GTV) encompassing the lesion. The planning target volume (PTV) covered the GTV with 5 mm margin. The treatment was performed with 6 megavoltage (MV) flattening filter free (FFF) photons on a linear accelerator using VMAT technique. (D) VMAT plan showing axial images of radiotherapy dose colour wash, with a hot spot (53.333 Gy) within the PTV.

Peritoneal progression occurred after these 7 months with 2 lesions in the peri-hepatic right dome and the right Morrison pouch detected on PET/CT imaging performed in December 2019. After multidisciplinary discussion, second line therapy consisting of Folfiri-Bevacizumab was offered to the patient. Since the disease was stable for 6 months, a cytoreductive surgery of the 2 PM was performed in May 2020. Pathology revealed 2 peritoneal metastases of colon primary adenocarcinoma (2 × 2 × 1.5 cm and 1 × 1 × 1 cm). A second local relapse involving both resected sites was found on the follow-up PET/CT: the peri-hepatic right dome (1.4 × 1 × 1.25 cm) and the right Morrison pouch (1 × 1.3 × 0.9 cm) was revealed on PET/CT imaging (Figures 1A and B and 2A and B). The second line therapy was rechallenged. After discussion at the multidisciplinary tumour board, the 2 PMs were considered as non-operable, and a stereotactic body radiation therapy (SBRT) was offered.

SBRT consisted of total dose of 48 Gy delivered in 6 fractions of 8 Gy given every other day to the PM in March 2021. The patient was simulated in supine position with deep inspiration breath hold (DIBH) to minimize doses to organ at risk (OAR). The gross tumour volume (GTV) definition of both metastases was guided by the PET/CT imaging and encompassed the lesion while excluding the liver and bone structures as far as they were not infiltrated. The planning target volume (PTV) covered the GTV with 5 mm margin (Figures 1C and 2C). The total dose 48 Gy in 6 fractions of 8 Gy was prescribed to PTV, according to ICRU 91 guidelines. Although the treatment was delivered in six fractions, planning quality was evaluated using target coverage objectives and OAR constraints recommended by AAPM Task Group 101 for five-fraction regimens. Small bowel hard constraints were set as Dmax < 35 Gy and V19.5 < 5 cc. Liver hard constraints were set as mean liver dose < 15 Gy. Spinal cord hard constraints were set as Dmax < 30 Gy.

The treatment was performed with 6 megavoltage (MV) flattening filter free (FFF) photons on a linear accelerator (TrueBeamSTX, Varian), using VMAT technique (Figures 1D and 2D). Before each fraction, image guidance with cone beam computed tomography (CBCT) was performed for accurate alignment of the target before treatment. Intra-fraction target motion was managed using DIBH condition and verified using CBCT images. Small bowel Dmax delivered to patient was 0.3 Gy and V19.5 was 0 cc. Mean Liver Dose delivered to patient was 2.6 Gy. Spinal cord Dmax delivered to patient was 3.7 Gy.

**Figure 3.**
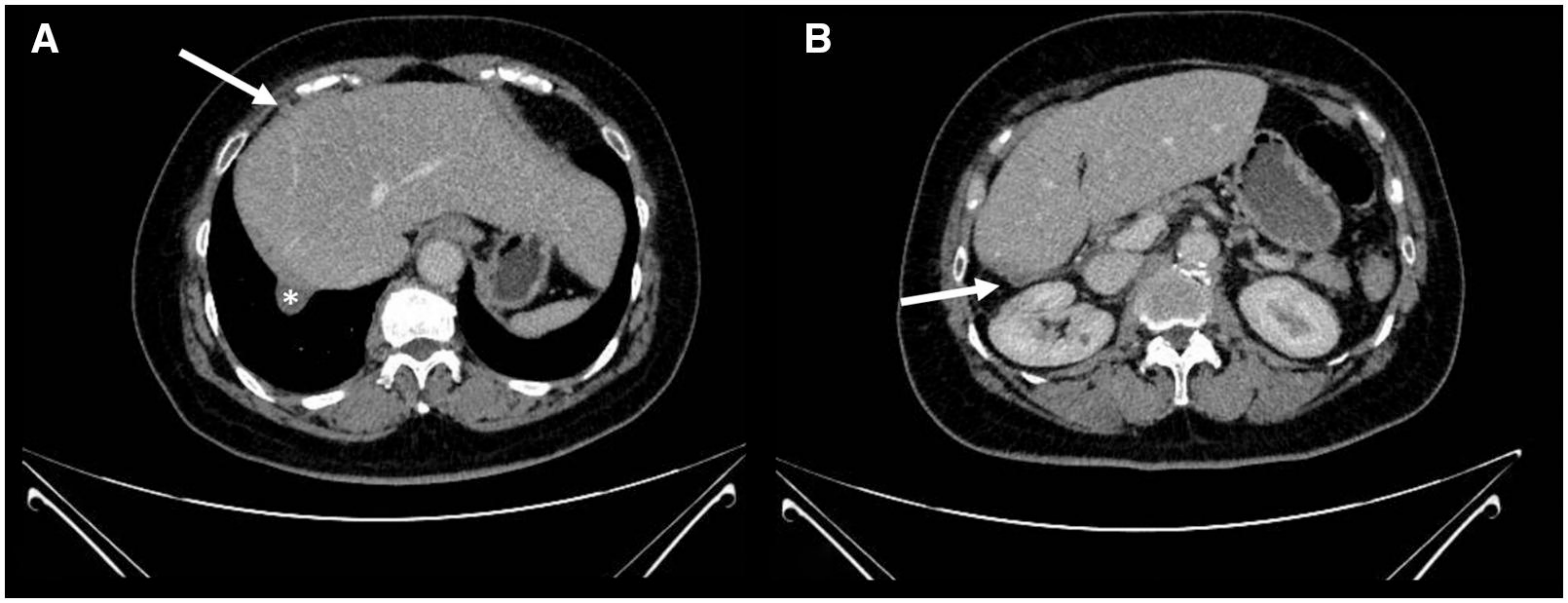
(A) Follow-up imaging (hepatic dome). CT-scan showing no recurrence in the treated area. The asterisk indicates the diaphragm. (B) Follow-up imaging (right Morrison pouch). CT-scan showing no recurrence in the treated area.

Treatment was delivered without interruptions. The tolerance of treatment was good. At 22 months of follow-up, no tumour regrowth or late complications (according to CTCAE 4.0 scale) were detected (Figure 3A and B).

Patient had stable disease on follow-up CT imaging, defined as the absence of significant lesion growth or new lesions. Complete disappearance of irradiated metastases is a rare outcome, and stable disease without progression is considered a favourable response in this setting.

## Discussion

PM is traditionally associated with associated with an extremely poor prognosis in metastatic colorectal cancer even with the advent of Oxaplatin and targeted therapies such as Bevacizumab and Cetuximab.^2^ The diagnosis of PM is itself challenging given their small size and limited contrast resolution resulting in underestimation of their real incidence and spread.^8^ Surgery remains the only curative option in a multimodal approach. Given the fact that only a proportion of patients are actually eligible for surgery for technical reasons or due to comorbidities, other ablative local therapies may be considered for oligometastatic PM.^2^

The early experience of radiation therapy using outdated techniques with whole abdominal irradiation (WAI) as the target volume was not satisfactory in terms of local control in the adjuvant and palliative setting for treating PM.^2^ Commonly used doses delivered for WAI treatments were 30 Gy in 1-1.5 Gy per fraction, with possibility of a boost of 16-20 Gy on primary site in compliant patients.^2^ These doses resulted respectively in a Biological Effective Dose of 34.5 Gy for α/β 10 (BED10) and 56.9 Gy for the boost (versus 86.4 Gy with 48 Gy in 6 fractions). However, the drastic improvement in radiation therapy delivery technology with the introduction of intensity modulated radiation therapy (IMRT) and stereotactic body radiation therapy (SBRT) along with IGRT (image guided radiation therapy) allows to deliver highly conformal treatment.^2^ SBRT is a non-invasive technique, which uses precise targeting to deliver ablative doses for small tumour targets while limiting dose to the surrounding organs.^2^ SBRT has rapidly developed in recent years thanks to its excellent locoregional control rate and high safety profile.^2^

Promising results have been described on SBRT for treatment of liver metastases in colorectal disease with 1-year local control (LC) >90% (range, 90%-95%), but there is scarce available data on PM in colorectal disease.^9^ For colorectal cancer, the ESMO recently defined oligometastatic disease as the involvement of up to 2, or occasionally 3 sites, including liver, lung, peritoneum, lymph nodes and ovary, with 5 or sometimes more metastases accessible to loco-regional treatment.^10^ There are also some limited reports of similar uses of SBRT for oligometastatic ovarian cancer.^11^

We identified in the literature 2 other patients with PM related to digestive cancer managed by SBRT (Table 1). The first had a native bifocal peritoneal metastasis from gastric cancer managed by 12 Gy single dose SBRT using VMAT technique.^12^ The second patient had a unique PM from colorectal cancer (as our case) managed by SBRT on an MRI-Linac, using intensity-modulated radiation therapy (IMRT) step and shoot technique.^2^ In this previous report, radiation dose and fractionation were based on intra-abdominal metastases treatment scheme (35 Gy in 5 fractions).^2^ Our report is unique in several aspects. Firstly, it is the first reported case of oligorecurrent PM from colorectal cancer managed by SBRT, using VMAT technique. In our case, we delivered 48 Gy in 6 fractions of 8 Gy, based on our institutional treatment scheme for liver metastases, resulting in a Biological Effective Dose of 86.4 Gy for α/β 10 (BED10) (versus 59.5 Gy with 35 Gy in 5 fractions and 26.4 Gy with 12 Gy in 1 fraction).^2^

**Table 1.** Cases of peritoneal metastases of digestive cancer treated with stereotactic body radiation therapy: review of literature.

Article	Number of cases	Symptoms	Treatment/radiation technique	RT dose/fractionation	Follow-up	Outcome
**Our case**	1	62-year-old woman *Disease*: Recurrent peritoneal metastasis. *Localization*: Right hepatic dome, right Morrison pouch. *Primary cancer*: Colorectal cancer *Indication*: Non-operable recurrence after surgery.	SBRT/VMAT	48 Gy in 6 fractions (8 Gy per fraction)	22 months	Stable disease. No recurrence. No notable complications.
**Boldrini et al^2^**	1	77-year-old woman *Disease*: Native peritoneal metastasis. *Localization*: Hypogastric-left iliac fossa area. *Primary cancer*: Colorectal cancer *Indication*: Non-operable metastasis.	SBRT/IMRT Step and Shoot technique.	35 Gy in 5 fractions (7 Gy per fraction)	10 months	Stable disease. No recurrence. No notable complications
**Mangel et al^12^**	1	58-year-old man *Disease*: Native peritoneal metastasis. *Localization*: Bifocal peritoneal metastases caudally from the edge of the liver and the left kidney. *Primary cancer*: Gastric cancer	SBRT/VMAT	12 Gy single dose	7 months	Stable disease. No recurrence. No notable complications

Abbreviations: IMRT = intensity-modulated radiation therapy; SBRT = stereotactic body radiation therapy; VMAT = volume-modulated arc therapy.

Main challenge in SBRT is to deliver very high doses into small target volumes in a highly accurate way.^7^ This can be achieved by using high-quality delivery techniques such as IMRT, VMAT, IGRT, and motion management.^2^^,^^7^ Peritoneal disease can be challenging to effectively treat with SBRT due to tumour locations often near gastro-intestinal (GI) luminal organs and the possibility of inter- and intra-fraction motion precluding effective and safe deliver. Due to the high variability of tumour variation within the bowel and the high risk of radiation induced bowel toxicity, only tumour with fixation to parietal peritoneum should be considered for SBRT. Several methods can be used to limit motion during treatment such as gating, DIHB, fiducial tracking, online adaptative radiotherapy.^2^ Our case was exceptionally favourable as the peritoneal disease was well distanced from GI luminal organs and likely was fixed on parietal peritoneum. MRI-Linac may be in closer future be more appropriate for more accurate treatment delivery by using fully online adaptative workflow.^2^

Interestingly, our patient was free of recurrence at 22-month follow-up. In the Boldrini et al^2^ and Mangel et al^12^ reports, both patients were in complete remission (range, 7-10 months). The development of novel markers predicting PM with high-risk accuracy is to be encouraged.^13^ Pitroda et al^14^ suggested the usefulness of integration of molecular subtyping to define patients with curative oligometastatic state in colorectal liver metastasis. In our case, pathologic features revealed subcapsular infiltration of the residual PM nodule. The early recurrence could be explained by potential tumour seeding. When surgery is indicated, caution must be made, and second-look surgery could help detecting early recurrence.^8^ It is to note, that our patient had no peritoneal chemotherapy. HIPEC is not contraindicated if SBRT is decided. SBRT could be a reasonable option in case of limited disease. In case of diffuse progression, SBRT is not reasonable. Due to the absence of synchronous metastases, her limited disease (2 PM), her age (>60 years), the lack of BRAF mutation, and the absence of mucinous or signet ring cell, with only localization in the proximal colon as a parameter associated with developing recurrent PM, we decided to treat her with a curative approach.^13^ Our case underlines the safety and possibility of curative approach in case of early, recurrent localized PM in digestive cancer. A 2020 report recently proposed a dynamic oligometastatic state model.^7^ This new model could help in accurate treatment decision. Oligometastatic disease could be divided into genuine oligometastatic disease (patient without previous polymetastatic disease) and into induced oligometastatic disease (patient with previous polymetastatic disease).^7^ Ablative treatment in curative intent could be offered in case of genuine oligometastatic disease (*de novo* oligometastatic disease or oligorecurrent disease), and local treatment should help the systemic treatment in case of induced oligometastatic disease, when the systemic treatment fails to destroy tumour cells.^7^ Despite the potential benefit of SBRT, surgery remains standard of care, allowing deep molecular analysis for personalized treatment. The comprehensive genomic profiling combined to the new dynamic oligometastatic state model is to be encouraged in case of recurrent PM disease to not omit a targetable alteration, select patients and to permit oriented treatment.

## Conclusion

Isolated peritoneal oligometastatic metastases are associated with poor prognosis. Cytoreductive surgery remains the historical standard of care treatment but represents a minority of all eligible patients. Diagnostic laparoscopic beforehand should be mandatory to rule out occult disease. Our patient had excellent local control (22-month follow-up) with no toxicity. Our case and few other reports suggest that stereotactic body radiation therapy is a safe, reasonable, and non-invasive option. SBRT has promising results in oligometastatic PM of colorectal cancer. MRI-Linac may be in closer future be more appropriate for more accurate treatment delivery by using fully online adaptative workflow. Despite the potential benefit of SBRT, surgery remains standard of care, allowing deep molecular analysis for personalized treatment.

## Learning points

- Peritoneal disease can be challenging to effectively treat with SBRT due to tumour locations often near GI luminal organs and the possibility of inter- and intra-fraction motion precluding effective and safe deliver.
- Due to the high propensity of tumour variation within the bowel and the high risk of radiation induced bowel toxicity, only tumour with fixation to parietal peritoneum should be considered for SBRT.
- Our case was exceptionally favourable as the peritoneal disease was well distanced from GI luminal organs and likely was fixed on parietal peritoneum, allowing SBRT to be delivered.
- SBRT resulted in excellent local control (22-month follow-up) with no toxicity.
- MRI-Linac may be in closer future be more appropriate for more accurate treatment delivery by using fully online adaptative workflow.

## Data Availability

The data that support the findings of this study are available from the corresponding author, Dr Alexander Bennassi, upon reasonable request.
